# A mechanism of growth inhibition by abscisic acid in germinating seeds of *Arabidopsis thaliana* based on inhibition of plasma membrane H^+^-ATPase and decreased cytosolic pH, K^+^, and anions

**DOI:** 10.1093/jxb/eru442

**Published:** 2014-11-04

**Authors:** María D. Planes, Regina Niñoles, Lourdes Rubio, Gaetano Bissoli, Eduardo Bueso, María J. García-Sánchez, Santiago Alejandro, Miguel Gonzalez-Guzmán, Rainer Hedrich, Pedro L. Rodriguez, José A. Fernández, Ramón Serrano

**Affiliations:** ^1^Instituto de Biología Molecular y Celular de Plantas, Universidad Politécnica de Valencia-Consejo Superior de Investigaciones Científicas, Camino de Vera, 46022 Valencia, Spain; ^2^Departamento de Biología Vegetal, Facultad de Ciencias, Universidad de Málaga, Campus de Teatinos, 29071 Málaga, Spain; ^3^Institute for Plant Physiology and Biophysics, University Würzburg, Julis-von-Sachs Platz 2, D-97082, Würzburg, Germany

**Keywords:** ABA receptors, cytosolic pH, ion channels, microelectrodes, protein kinase, proton efflux.

## Abstract

The stress hormone abscisic acid (ABA) induces expression of defence genes in many organs, modulates ion homeostasis and metabolism in guard cells, and inhibits germination and seedling growth. Concerning the latter effect, several mutants of *Arabidopsis thaliana* with improved capability for H^+^ efflux (*wat1-1D*, overexpression of *AKT1* and *ost2-1D*) are less sensitive to inhibition by ABA than the wild type. This suggested that ABA could inhibit H^+^ efflux (H^+^-ATPase) and induce cytosolic acidification as a mechanism of growth inhibition. Measurements to test this hypothesis could not be done in germinating seeds and we used roots as the most convenient system. ABA inhibited the root plasma-membrane H^+^-ATPase measured *in vitro* (ATP hydrolysis by isolated vesicles) and *in vivo* (H^+^ efflux from seedling roots). This inhibition involved the core ABA signalling elements: PYR/PYL/RCAR ABA receptors, ABA-inhibited protein phosphatases (HAB1), and ABA-activated protein kinases (SnRK2.2 and SnRK2.3). Electrophysiological measurements in root epidermal cells indicated that ABA, acting through the PYR/PYL/RCAR receptors, induced membrane hyperpolarization (due to K^+^ efflux through the GORK channel) and cytosolic acidification. This acidification was not observed in the *wat1-1D* mutant. The mechanism of inhibition of the H^+^-ATPase by ABA and its effects on cytosolic pH and membrane potential in roots were different from those in guard cells. ABA did not affect the *in vivo* phosphorylation level of the known activating site (penultimate threonine) of H^+^-ATPase in roots, and SnRK2.2 phosphorylated *in vitro* the C-terminal regulatory domain of H^+^-ATPase while the guard-cell kinase SnRK2.6/OST1 did not.

## Hghlight

We provide the first evidence for a mechanism of growth inhibition by ABA during germination and seedling establishment based on inhibition of PM H^+^-ATPase and altered pH, K+, and anion homeostasis.

## Introduction

The hormone abscisic acid (ABA) plays a critical role in plant stress responses by transcriptional induction of defence genes in different organs. During the closing of stomata, however, ABA has a non-transcriptional mechanism modulating ion homeostasis and resulting in inhibition of plasma membrane (PM) H^+^-ATPase, membrane depolarization, cytosolic alkalinisation, and efflux of K^+^ and anions (Fujii *et al.*, 2009; Kim *et al.*, 2010; Joshi-Saha *et al.*, 2011). Recent results in *Arabidopsis thaliana* (*Arabidopsis*) indicate that the same core signal transduction pathway operates in both cases, with ABA bound to PYR/PYL/RCAR (PYRABACTIN RESISTANCE1 /PYR1-LIKE/ REGULATORY COMPONENTS OF ABA RECEPTORS; hereafter referred to as PYR/PYL) receptors inhibiting clade A of PP2C protein phosphatases (ABI1, ABI2, HAB1, and PP2CA) and resulting in activation of a subgroup of SnRK2 protein kinases (2.2, 2.3 and guard-cell OST1/2.6). In the transcriptional mechanism, these kinases phosphorylate and activate a subgroup of bZIP transcription factors including ABI5 (Lopez-Molina *et al.*, 2002) and ABFs/AREBs (Kang *et al.*, 2002; Yoshida *et al.*, 2010) that recognize the ABRE promoter element (consensus PyACGTGG/TC) in ABA-responsive genes (Fujii *et al.*, 2009; Joshi-Saha *et al.*, 2011). In the ion homeostasis mechanism of guard cells, the kinases phosphorylate and regulate ion transporters such as suggested for the K^+^ uptake channel KAT1 (inhibited; Sato *et al.*, 2009), the K^+^ efflux channel GORK (activated; Ache *et al.*, 2000; Hosy *et al.*, 2003), and the anion efflux channel SLAC1 (activated; Geiger *et al.*, 2009). The PM H^+^-ATPase of guard cells is inhibited by ABA through a pathway involving the kinase OST1/SnRK2.6 (Merlot *et al.*, 2007). This results in dephosphorylation of the activating site of the PM H^+^-ATPase (penultimate threonine-947; Yin *et al.*, 2013) by an unknown mechanism.

In addition to induction of stress defence genes and closing of stomata, one important function of ABA is to inhibit germination and early seedling growth. A transcriptional mechanism dependent on the seed-specific transcription factor ABI5 (Lopez-Molina *et al.*, 2002; Piskurewicz *et al.*, 2008) and resulting in inhibition of the cell cycle (Wang *et al.*, 1998) and metabolism (Penfield *et al.*, 2006) has been proposed. In addition, the activities of the PM H^+^-ATPase (Haruta and Sussman, 2012) and of nutrient transporters (especially K^+^ channels) coupled to the electrochemical proton gradient are important for growth (Sano *et al.*, 2007) and could be modulated by ABA. Therefore, a mechanism of growth inhibition based on altered ion homeostasis could also be operative.

Several mutants of *Arabidopsis* with improved capability for H^+^ efflux in roots are more tolerant to inhibition of growth by weak organic acids during germination and early seedling growth. These include *wat1-1D* and overexpression (OE) of *AKT1* (Niñoles *et al.*, 2013) and *ost2-1D* (Merlot *et al.*, 2007). These acids induce cytosolic acidification (Niñoles *et al.*, 2013) and our work started with the observation that acid-tolerant mutants are less sensitive to inhibition of germination and seedling establishment by ABA than the wild type. We formulated the hypothesis that one mechanism of inhibition of growth by ABA at this early stage is the impairment of pH homeostasis (cytosolic acidification) through inhibition of PM H^+^-ATPase. As the experiments to test this hypothesis could not be done in germinating seeds, we decided to use roots as the most convenient system because in previous works biochemical and electrophysiological measurements in roots correlated with growth phenotypes in germinating seeds (Bissoli *et al.*, 2012; Niñoles *et al.*, 2013).

The effects of ABA on ion homeostasis of roots have not been investigated in detail (Pilot *et al.*, 2003; Hedrich, 2012). There are indications that differences exist between guard and root cells (Roberts and Snowman, 2000), but no measurements of cytosolic pH and ion concentrations have been made in the latter. On the other hand, the effect of ABA on the root PM H^+^-ATPase is controversial. In tomato roots, salt stress decreases the activity of the H^+^-ATPase (Gronwald *et al.*, 1990). while in cucumber roots, salt stress and ABA increase the activity and expression of the enzyme (Janicka-Russak and Klobus, 2007).

In the present work, we took advantage of *Arabidopsis* mutants in the ABA signal transduction pathway and ion channels to investigate the mechanism of the effects of ABA on the activity of root PM H^+^-ATPase and on cytosolic pH, [K^+^], and [Cl^–^] of root epidermal cells. We found that ABA acted in roots through the core signal transduction pathway described above and that it inhibited the root PM H^+^-ATPase and induced cytosolic acidification in root epidermal cells. These results support a mechanism of growth inhibition by ABA in germinating seeds of *Arabidopsis* based on inhibition of PM H^+^-ATPase and cytosolic acidification. This ion homeostasis mechanism could operate upstream of the transcriptional mechanism dependent on ABI5.

## Materials and methods

### Plant material and growth conditions

*Arabidopsis* seeds were stratified for 3 d at 4 °C and germinated and grown on Murashige and Skoog (MS) plates with 1% agar, 1% sucrose and 10mM 2-[*N*-morpholino]ethanesulfonic acid (MES) adjusted to pH 5.7 with Tris base. The plates were sealed and incubated in a growth chamber at 22 °C under 16h light (80–100 µE m^–2^ sec^–1^) and 8h dark. For propagation under greenhouse conditions, pots contained a 1:2 vermiculite:soil mixture and were irrigated with a modified Hoagland mineral nutrient solution (Naranjo *et al.*, 2003). For root production, a hydroponic culture system was utilized (Araponics, Liège, Belgium) with the same nutrient solution described above and under short-day conditions (8h light/16h dark).

The following genotypes of *Arabidopsis* were utilized: wild-type ecotype Columbia-0 and derived mutants *wat1-1D* and OE *AKT1* (overexpression of *AKT1*; Niñoles *et al.*, 2013), *pyr1 pyl1 pyl2 pyl4 pyl5 pyl8* (*112458 pyr/pyl*; Gonzalez-Guzmán *et al.*, 2012), OE *HAB1* (overexpression of *HAB1*; Saez *et al.*, 2004), *snrk2.2 snrk2.3* (Fujii *et al.*, 2007), and slah3-1 (Gutermuth *et al.*, 2013); wild-type ecotype Landsberg erecta and derived mutant *ost2-1D* (Merlot *et al.*, 2007); and wild-type ecotype Wassilevskija (WS 0) and derived *gork1-1* mutant (Hosy *et al.*, 2003).

### Purification of PM vesicles from roots and determination of PM H^+^-ATPase activity

*Arabidopsis* plants were grown hydroponically for 2 months and 0.2–0.3g of roots per plant was obtained. Purification of PM vesicles was done with about 2g of roots. The method described by Serrano (1988) for oat roots was followed as modified by Niñoles *et al*. (2013). Briefly, homogenization was effected with a mortar and pestle after freezing with liquid nitrogen, and purification was carried out by sucrose gradient centrifugation with two layers of sucrose gradient of 41 and 33% sucrose (w/w). The final yield was 0.2–0.3mg of protein. PM H^+^-ATPase activity was determined at pH 6.5 as the ATP hydrolysis activity sensitive to 50 µM diethylstilbestrol (Serrano, 1988; Niñoles *et al.*, 2013) and represented more than 80% of the ATP hydrolytic activity in the preparations. Protein concentration was determined by the method of Bradford (1976).

### Determination of proton efflux from roots

Two external acidification assays were used as described by Bissoli *et al.* (2012). Briefly, plants were grown on vertical plates with normal MS medium for 14 d. For the solid medium assay, plants were transferred to MS vertical plates without buffer and with 0.003% bromocresol purple pH indicator and incubated in the light for 6–8h. For the liquid medium assay, plants were transferred to a plate with sterile water and incubated for 24h in the dark. The starved plants were incubated in a vessel with MS medium without buffer (only the roots were submerged), and proton efflux was started by addition of sucrose (1% final, time 0). Acidification was recorded with a sensitive pH meter (Crison GLP22 with pH electrode 52.08; Alella, Barcelona, Spain).

### Electrophysiological measurements of membrane potential, cytosolic pH, K^+^ and Cl^–^, and external K^+^ and Cl^–^ in young roots

Membrane potential and cytosolic pH were measured using double-barrelled microelectrodes with one of their bars containing a H^+^-specific sensor (ETH1907) as described previously (Fernández *et al.*, 1999; Bissoli *et al.*, 2012; Niñoles *et al.*, 2013). Plantlets of 2 weeks old with roots of about 0.5cm were used. H^+^-selective microelectrodes were calibrated before and after the measurement using buffer solutions containing 96mM KCl and 2.5mM MES/Bis/Tris propane (pH 5.3, 6.3, 7.3, and 8.3). The calibration curves showed slopes around 45 mV per pH unit.

To measure cytosolic K^+^, double-barrelled microelectrodes were pulled and sylanized as pH microelectrodes but backfilled with K^+^ ionophore I sensor (Fluka) as described previously (Mithöfer *et al.*, 2005; Barragan *et al.*, 2012). K^+^-selective microelectrodes were calibrated with different KCl solutions, from 1 to 100mM KCl, showing slopes of approximately 45 mV per pK^+^. Seedlings were grown vertically in MS medium for 10–15 d, the roots mounted in a perfusion chamber, and the microelectrodes inserted into epidermal cells. Glass for chloride microelectrodes was pulled and sylanized as indicated for the pH and K^+^ microelectrodes but was filled with the chloride ionophore I (cocktail A) (Fluka). Chloride microelectrodes were calibrated before and after the measurements against NaCl standard solutions containing 5mM NaNO_3_, the putative cytosolic NO-_3_ concentration (Miller and Smith, 2008). Calibration curves show typically a slope of 38 mV per pCl. The assay medium contained 0.1mM NaCl, 0.1mM KCl, 0.1mM CaCl_2_, and 12mM MES adjusted to pH 5.7 with Bis/Tris propane. ABA was added at 10 µM and the solutions were stable for at least 30min.

To measure external K^+^ and Cl^–^, single-barrelled electrodes of 1.5mm of external diameter, with internal filament, were pulled in a patch clamp puller in order to get an open tip of 2 µm external diameter. Once the capillaries were pulled, they were sylanized and backfilled with the appropriate ionic sensor. Electrode tips were placed 5 µm out from the root epidermis.

### Western blot analysis of the PM H^+^-ATPase

Quantification of the PM H^+^-ATPase by western blotting was made with a rabbit antibody raised against the C-terminal domain of the AHA3 isoform of *Arabidopsis* (Parets-Soler *et al.*, 1990). This domain (last 98 aa) is highly conserved (more than 90% identity) between the three major isoforms (AHA1, -2, and -3; Harper *et al.*, 1990) and therefore it is unlikely that the antibody could differentiate between them.

The antibody against the phosphorylated peptide of the known activating site of AHA2 H^+^-ATPase (last 9 aa, penultimate Thr947 phosphorylated) was obtained from Toshinori Kinoshita (Nagoya University, Japan) and has been described elsewhere (Hayashi *et al.*, 2010, 2011). This peptide is fully conserved between major isoforms in the last 4 aa (HYpTV) and the antibody cross-reacts with all major phosphorylated isoforms (Hayashi *et al.*, 2011).

Detection of the labelled blots was done using an Amersham ECL system (GE Heathcare Life Sciences, Buckinghamshire, UK) and quantification was done with the Java-based image-processing program ImageJ (http://rsb.info.nih.gov/ij/). Blots were first decorated with the antibody against the phosphorylated peptide described above, stripped by incubation for 1h in 0.2M glycine/HCl (pH 2.7), decorated with antibody against the H^+^-ATPase described above, and finally stained for total protein with Direct Blue 71.

### Production of the recombinant C-terminal domain of AHA2 H^+^-ATPase and of OST1/SnRK2.6 in bacteria

A cDNA of AHA2 H^+^-ATPase (Pardo and Serrano, 1989) was amplified with primers AHA2Ct-F and AHA2Ct-R (Supplementary Table S1 at *JXB* online) resulting in a fragment of 323bp encoding the last 106 aa of the protein, from R842 to V948. It was cloned into the pMAL-c2 expression vector (New England Biolabs, MA, USA) behind and in frame with maltose-binding protein (MBP). This construct was utilized as a template to generate point mutations by overlap-extension PCR (Patel *et al.* 2009). The primers for the mutations S899P, S904L, S931F, and T924A are shown in Supplementary Table S1. As indicated, all were designed to introduce a new restriction site. To obtain the double mutant S931F T924A, the plasmid with the S931F mutation was used as template to introduce the second mutation. All mutations were verified by sequencing. The recombinant fusion proteins (MBP–CtAHA2) were purified by amylose affinity chromatography following the instructions of the vector manufacturer.

The purification of recombinant His-OST1/SnRK2.6 was as described by Fujii *et al.* (2009).

### Generation of haemagglutinin (HA)-tagged SnRK2.2 transgenic *Arabidopsis* lines

The coding sequence of SnRKs2.2 (Fujii *et al.*, 2007) was amplified by PCR using the primer pair F3g50500 and R3g50500 (Supplementary Table S1). The PCR products were cloned into pCR8/GW/TOPO (Invitrogen, CA, USA) following the instructions of the manufacturer. After sequencing verification, coding sequences in the pCR8/GW/TOPO entry clone were recombined by LR reaction into the Gateway-compatible ALLIGATOR2 vector (Bensmihen *et al.*, 2004). The ALLIGATOR2 vector drives expression of the recombined gene under control of the 35S cauliflower mosaic virus promoter and introduces a triple HA epitope at the N terminus of the encoded protein. Generation and selection of SnRK2.2-overexpresing transgenic lines was done as described previously (Dupeux *et al.*, 2011). Homozygous T3 progeny was used for further studies, and expression of HA-tagged protein was verified by western blot analysis using horseradish peroxidase-conjugated anti-HA (Roche Applied Science, Penzberg, Germany).

### Inmunoprecipitation of 3HA–SnRK2.2 and *in vitro* phosphorylation of MBP–CtAHA2 by 3HA–SnRK2.2

HA-tagged SnRK2.2 protein was obtained by immunoprecipitation from 35S:HA–SnRK2.2 transgenic lines. Ten surface-sterilized 5-d-old seedlings grown on MS plates supplemented with 1% sucrose were transferred to 100ml flasks containing 2.5ml of MS medium plus 1% sucrose in a controlled-environment growth chamber. After 15 d, the seedlings were treated with 100 μM ABA for 30min and plant material was collected and frozen in liquid nitrogen. Plant material (0.5g) was extracted in 2 vols of 1× PBS supplemented with 1mM EDTA, 0.05% Triton X-100, 1/500 (v/v) plant-specific protease inhibitor cocktail (Sigma), 5mM dithioreitol, 10mM NaF, 1mM PMSF, and 10 μM ABA. After centrifugation (16 000*g*, 4°C, 15min), the supernatant was recovered, and 1mg of total protein was incubated overnight at 4 °C with 25 μl of anti-HA affinity matrix (Roche Applied Science). The matrix was washed three times with extraction buffer and two more times with kinase buffer (10mM Tris/HCl, pH 7.8, 10mM MgCl, 0.5mM dithioreitol, and 2mM MnCl_2_). Finally, the matrix was resuspended in a total volume of 50 μl in kinase buffer, of which 4 μl was used for each reaction.

For *in vitro* kinase assays, immunoprecipitated SnRK2.2 was incubated with 0.5 µg of 6×His–ΔCABF2, MBP, MBP–ABA2 (Gonzalez-Guzmán *et al.*, 2002), wild-type MBP–CtAHA2, or mutated MBP–CtAHA2 in 30 μl of kinase buffer for 1h at room temperature in the presence of 3.5 μCi of [γ^32^P]ATP. The reaction products were resolved in an 8% SDS-PAGE gel, transferred to an Immobilon-P membrane (Merck Millipore, Darmstadt, Germany), and detected using a phosphorimager system (FLA5100; Fujifilm, Tokyo, Japan).

### Quantification of mRNAs by reverse transcription real-time PCR

RNA free of DNA was prepared by homogenization of tissue frozen with liquid nitrogen using a mortar and pestle, extraction with guanidine thiocyanate, and purification by binding to a silica gel porous plate, treatment with DNAse, and elution using a NucleoSpin RNA kit (Macherey-Nagel, Düren, Germany). Reverse transcription was done with a Maxima First Strand cDNA Synthesis kit for RT-qPCR (Thermo Scientific, Massachusetts, USA) and for quantitative real-time PCR we utilized a 5× PyroTaq EvaGreen qPCR Mix Plus (ROX) kit (Cultek Molecular Bioline, Madrid, Spain). The primers for detection of ABI5 (ABI5-F and ABI5-R) and COR78/29A (COR78-f and COR78-R) as well as the primers for the reference genes ACT8 (ACT8-F and ACT8-R) and PP2AA3 (PP2AA3-F and PP2AA3-R) (Czechowski *et al.*, 2005) are shown in Supplementary Table S1. Primer products were about 0.2kb and we used a 7500 Fast Real-Time PCR System (Applied Biosystems, CA, USA).

## Results

### Germination and early seedling growth of *Arabidopsis* mutants with improved pH homeostasis are less sensitive to ABA

**Fig. 1.**
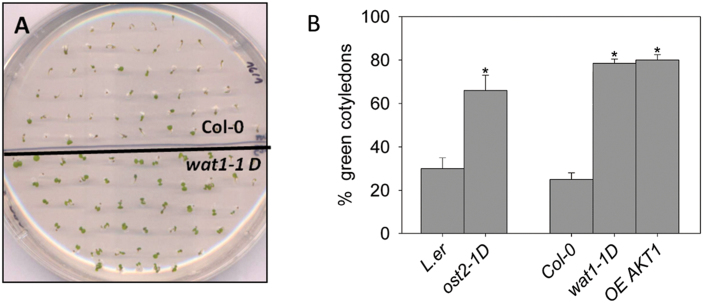
Germination and early seedling growth of *Arabidopsis* mutants *wat1-1D*, OE *AKT1*, and *ost2-1D* are less sensitive to inhibition by ABA than in the wild type. (A) Visual result of a typical experiment with Col-0 and *wat1-1D*. (B) statistical data from three independent experiments. Values are the average percentages of seedlings with green, expanded cotyledons (reflecting seedling establishment) after 7 d in plates with 0.75 µM ABA. In the absence of ABA, 95–100% of planted seeds had green cotyledons in all genotypes. Two independent lines of OE *AKT1* gave similar results. The mutants *wat1-1D* and OE *AKT1* were in the Columbia (Col-0) background, while *ost2-1D* was in the Landsberg erecta (L.er) background. Error bars correspond to the standard error. * indicates a significant difference (*P*<0.01 by Student’s *t*-test) compared with wild type. (This figure is available in colour at *JXB* online.)

Several mutants with improved pH homeostasis because of increased capability for proton extrusion (as determined in roots) have been identified in *A. thaliana.* These include *ost2-1D* (a constitutively hyperactive plasma membrane AHA1 H^+^-ATPase; Merlot *et al.*, 2007), *wat1-1D* (loss of function of AP-3 β-adaptin affecting localization of transporters; Niñoles *et al.*, 2013) and the overexpression of the K^+^ uptake channel *AKT1* (OE *AKT1*; Niñoles *et al.*, 2013). In *wat1-1D* and OE *AKT1*, an increased rate of K^+^ influx allows a higher rate of H^+^ efflux mediated by the PM H^+^-ATPase through electrical balance. These mutants are tolerant of inhibition of germination and early seedling growth by weak organic acids such as acetic acid, which induces cytosolic acidification in root epidermal cells of the wild type but not in the *wat1-1D* mutant (Niñoles *et al.*, 2013). As indicated in Fig. 1, all these mutants were less sensitive than the wild type to inhibition by ABA during germination and early seedling growth (appearance of green cotyledons). These results suggested that one mechanism of inhibition of growth by ABA at this stage could be cytosolic acidification. The ABA insensitivity of the *wat1-1D* mutant was much less apparent during plantlet growth (Supplementary Fig. S1 at *JXB* online).

### ABA inhibits root PM H^+^-ATPase and H^+^ efflux

In order to obtain evidence for the postulated cytosolic acidification induced by ABA in germinating seeds, biochemical determinations of PM H^+^-ATPase and H^+^ and K^+^ fluxes and electrophysiological measurements of cytosolic pH and K^+^ need to be made. These experiments, however, are difficult in germinating seeds and therefore we used roots as a surrogate, a strategy utilized in previous studies (Bissoli *et al.*, 2012; Niñoles *et al.*, 2013). In the electrophysiological experiments, small roots (about 0.5cm) of plantlets were used, a material not very different from radiculas.

**Fig. 2.**
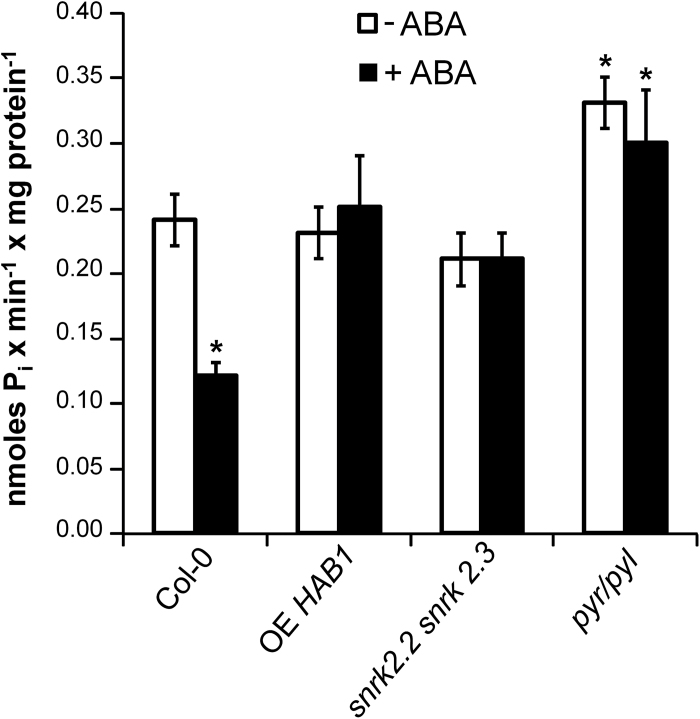
Effect of ABA on the activity of PM H^+^-ATPase from roots of different *Arabidopsis* genotypes. Plants were grown in hydroponic culture, treated or not for 2h with 10 µM ABA, the PMs were purified from roots, and diethylstilbestrol-sensitive ATP hydrolysis by the H^+^-ATPase was determined as indicated in Materials and methods. The wild type was Columbia-0 (Col-0). The specific activity was expressed in nmoles *P*_i_ min^–1^ mg protein^–1^ and values are the average of three to five independent experiments±standard error. White bars: control conditions; black bars: ABA treatment. * indicates a significant difference (*P*<0.01) compared with the activity of the wild type without ABA treatment (Student’s *t*-test). OE *HAB1*, OE mutant of protein phosphatase gene *HAB1*; *snrk2.2 snrk2.3*, double mutant in protein kinases *snrk2.2 snrk2.3*; *pyr/pyl*, sextuple knockout mutant 112458 *pyr/pyl*.

Treatment of *Arabidopsis* wild-type plants in hydroponic culture with 10 µM ABA, a concentration producing maximal inhibitory effects in growth studies (Gonzalez-Guzmán *et al.*, 2012), resulted in 50% inhibition of root PM H^+^-ATPase activity as determined by diethylstilbestrol-sensitive ATP hydrolysis in purified plasma membranes (Fig. 2). This inhibition was not observed in ABA-insensitive mutants such as OE of protein phosphatase gene *HAB1* (Saez *et al.*, 2004), the double mutant in protein kinases *snrk2.2 snrk2.3* (Fujii *et al.*, 2007) and the sextuple *112458 pyr/pyl* in ABA receptors (Gonzalez-Guzmán *et al.*, 2012), suggesting that it is mediated by the known ABA signal transduction pathway. SnRK2.2 and SnRK2.3 are the two redundant ABA-activated protein kinases expressed in tissues different from guard cells, which express OST1/SnRK2.6 (Fujii *et al.*, 2007). Interestingly, the *112458 pyr/pyl* mutant exhibited about 30% higher PM H^+^-ATPase activity in the absence of ABA than the wild type. After ABA treatment, its PM H^+^-ATPase activity was 2.5-fold higher than that of the wild type (Fig. 2).

**Fig. 3.**
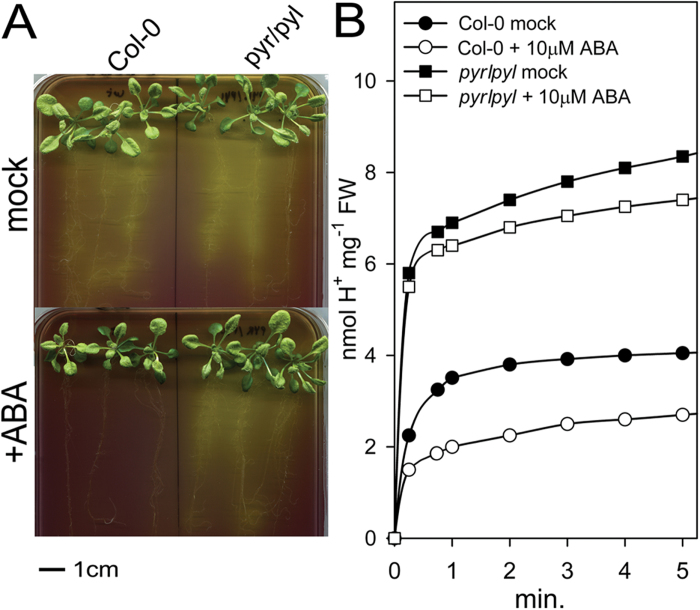
Inhibition by ABA of H^+^ efflux from roots of *Arabidopsis* wild type (Col-0) and sextuple knockout of ABA receptors *112458 pyr/pyl* (*pyr/pyl*). (A) Qualitative assay on plates with the pH indicator bromocresol purple. The result of a typical experiment is shown. A yellow colour around the roots after 8h indicates proton extrusion, and similar results were obtained in three independent experiments. (B) Quantitative determination of H^+^ efflux from plant roots. Sucrose (1%) was added to starved plants at time 0. Circles: wild type; squares: *pyr/pyl* mutant; closed symbols: control conditions; open symbols: 10 µM ABA added 10min before time 0. Values are means of three repetitions and standard errors (not shown for clarity) were 0.3–0.5 (Col-0, circles) and 0.6–1 (*pyr/pyl*, squares) nmol H^+^ mg^–1^ of fresh weight (FW) of roots. (This figure is available in colour at *JXB* online.)

This inhibition of the PM H^+^-ATPase by ABA correlated with inhibition of H^+^ extrusion from roots measured in solid medium with the pH-sensitive dye bromocresol purple (Fig. 3A) and in liquid medium with a sensitive pH meter (Fig. 3B). In the ABA-insensitive *112458 pyr/pyl* mutant, the inhibition by ABA was much less apparent in both solid and liquid media, and the H^+^ extrusion activity in the absence of ABA was greater than in the wild type. This correlated with the increased PM H^+^-ATPase activity described above, although the effect was more apparent in H^+^ extrusion (Fig. 3B). This assay, however, suffered from the lack of true initial rates because technical problems preclude taking measurements at times shorter than 15 s. This problem may explain why a modest 30% increase of PM H^+^-ATPase activity of the *112458 pyr/pyl* mutant in control conditions (Fig. 2) translated into an approximately 100% increase in H^+^ extrusion (Fig. 3B).

### SnRK2.2 phosphorylates the regulatory domain of AHA2 H^+^-ATPase

The PM H^+^-ATPase is a highly regulated enzyme, with blue light (Kinoshita and Shimazaki, 1999; Hayashi *et al.*, 2011), auxin (Hager, 2003; Takahashi *et al.*, 2012), intracellular acidification (Bobik *et al.*, 2010), sucrose metabolism (Niittylä *et al.*, 2007), and acetylated 1,3-diaminopropane (Jammes *et al.*, 2014) triggering activation of the enzyme, while extracellular alkalinization (Fuglsang *et al.*, 2007) and ABA in guard cells (Merlot *et al.*, 2007; Hayashi *et al.*, 2011; Yin *et al.*, 2013) and hypocotyls (Hayashi *et al.*, 2014) inhibit the enzyme. Activation results from phosphorylation of the penultimate threonine (which in *Arabidopsis* is Thr948 in the AHA1 isoform and Thr947 in the AHA2 isoform) by an unknown kinase and binding of 14-3-3 proteins (Olsson *et al.*, 1998; Fuglsang *et al.*, 1999; Kinoshita and Shimazaki, 1999). In guard cells and hypocotyls, phosphorylation of the penultimate threonine is decreased by ABA by an unknown mechanism (Hayashi *et al.*, 2011; Yin *et al.*, 2013; Hayashi *et al.*, 2014). A recently proposed mechanism for auxin activation is the induction of SAUR proteins that inhibit clade D PP2C phosphatases to keep the penultimate threonine of H^+^-ATPase phosphorylated (Spartz *et al.*, 2014).

In our experimental system of *Arabidopsis* roots, western blot analysis indicated that neither the level of root PM H^+^-ATPase protein nor phosphorylation of the known activating site of the AHA2 enzyme (Thr947) were decreased by ABA (Supplementary Fig. S2 at *JXB* online). In fact, quantification of the band areas indicated that ABA treatment induced a small increase of ATPase protein and of the Thr947 phosphorylated form.

**Fig. 4.**
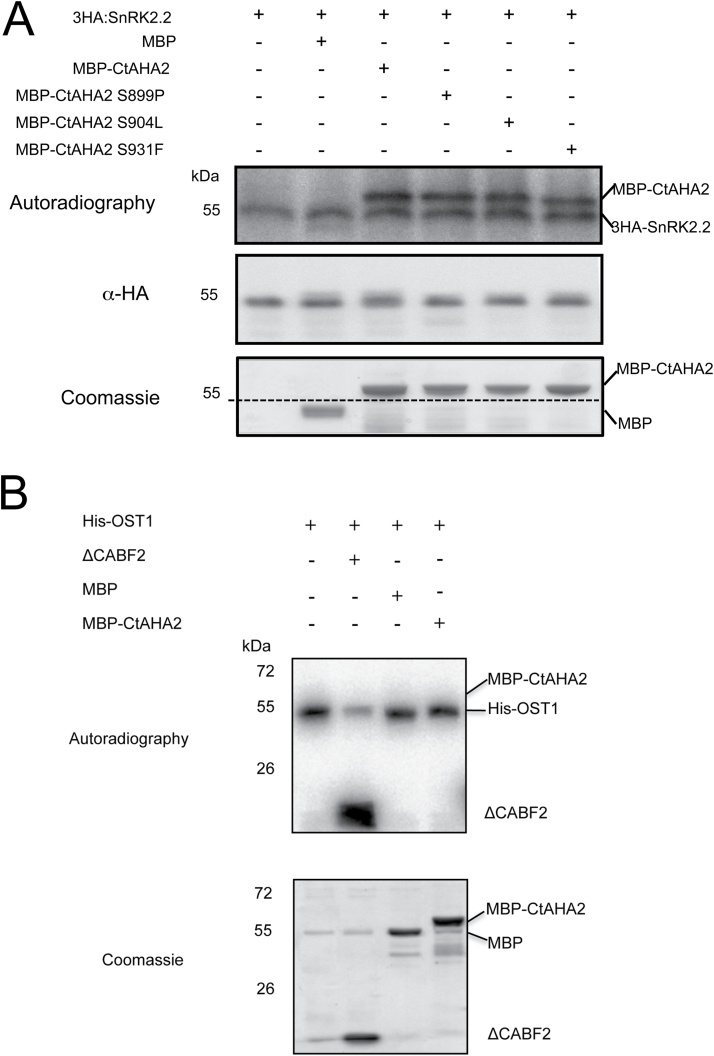
SnRK2.2 specifically phosphorylates the C-terminal regulatory domain (Ct) of AHA2 PM H^+^-ATPase *in vitro*. (A) 3HA–SnRK2.2, MBP–CtAHA2, and MBP were purified and added to the kinase assay as indicated. Autophosphorylation of SnRK2.2 and phosphorylation of CtAHA2 was observed by autoradiography. MBP was not phosphorylated. The mutated versions of the C-terminal fragment (S899P, S904L, or S931F) were phosphorylated as efficiently as the wild type. The dashed line in the Coomassie staining panel indicates the migration of the immunoprecipitated 3HA–SnRK2.2, which was estimated by overlapping with the anti-HA western blot. (B) His–OST1/SnRK2.6, ΔCABF2, MBP, and MBP–CtAHA2 were purified and added to the kinase assay as indicated. Autophosphorylation of His-OST1/SnRK2.6 and phosphorylation of ΔCABF2 was observed by autoradiography. Neither MBP nor MBP–CtAHA2 were phosphorylated. The experiments were repeated twice with similar results.

An alternative mechanism of regulation could be ABA-induced phosphorylation of inhibitory sites within the regulatory C-terminal domain of the enzyme, such as Ser931 (Fuglsang *et al.*, 2007) and Ser899 (Haruta *et al.*, 2014). As indicated in Fig. 4A (lane 3), the ABA-activated protein kinase SnRK2.2 phosphorylated the C-terminal regulatory domain of AHA2 *in vitro*. This could not result from non-specific phosphorylation because this kinase could not phosphorylate either MBP (Fig. 4A, lane 2) or the enzyme ABA2 (Supplementary Fig. S3, lane 3 at *JXB* online) but it did phosphorylate itself (autophosphorylation) and its physiological substrate ABF2 (Supplementary Fig. S3, lane 1). Another piece of evidence against non-specific phosphorylation in our assay is provided in Fig. 4B: the guard-cell protein kinase OST1/SnRK2.6 could phosphorylate ABF2 (substrate of all ABA-activated kinases) but not the C-terminal regulatory domain of AHA2.

We mutagenized to non-phosphorylable amino acids several candidate phosphorylation sites (Ser904, Thr924, and Ser931) according to the known specificity of SnRK protein kinases (RXXS/T), as well as another potential site, Ser899 (Joshi-Saha *et al.*, 2011; Rudashevskaya *et al.*, 2012). These single mutations as well and the double mutation on T924A and S931F were without effect on the *in vitro* phosphorylation of the C-terminal domain of AHA2 ATPase by SnRK2.2 (Fig. 4 and Supplementary Fig. S3).

### ABA induces cytosolic acidification in root epidermal cells

**Fig. 5.**
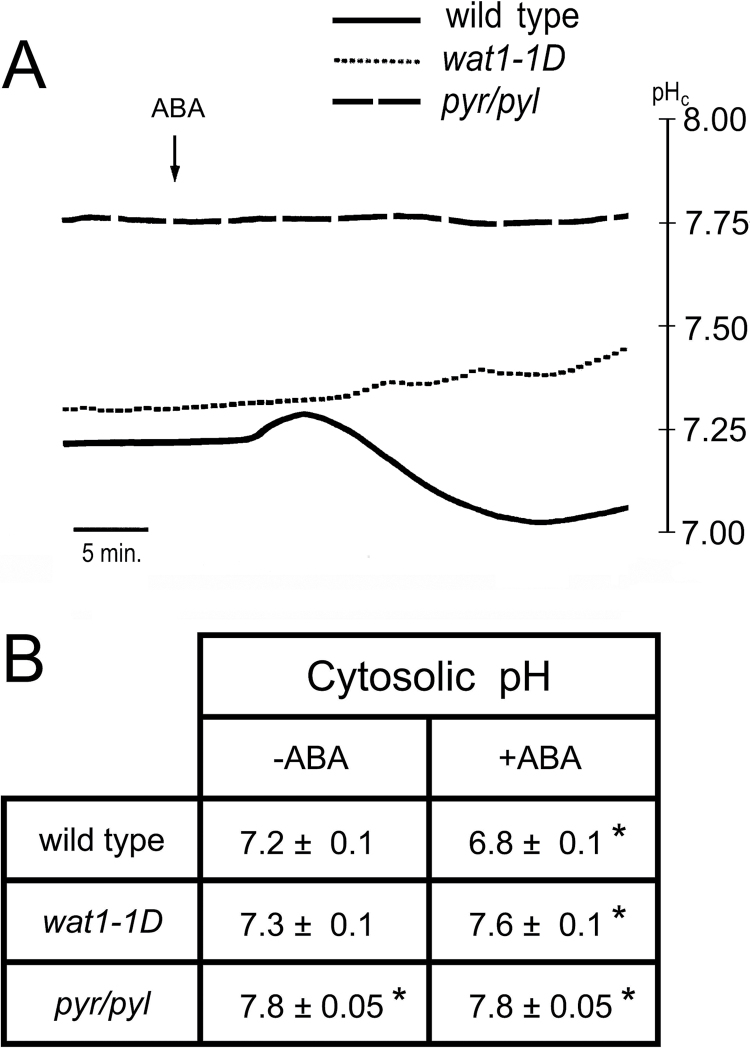
Effect of ABA on cytosolic pH (pH_c_) of root epidermal cells measured with microelectrodes in wild type Col-0, the *wat1-1D* mutant, and the sextuple knockout in ABA receptors mutant *112458 pyr/pyl* (*pyr/pyl*). (A) Time course of a typical experiment with wild type (continuous line), *wat1-1D* (short dashes), and *pyr/pyl* (long dashes). (B) Statistical data from four to six independent experiments as in (A). The average cytosolic pH±standard error is given. * indicates significant difference (*P*<0.05 by Student’s *t*-test) compared with wild type without ABA.

As the PM H^+^-ATPase is the major proton extrusion pump of plant cells (Gaxiola *et al.*, 2007; Duby and Boutry, 2009; Haruta and Sussman, 2012), its inhibition by ABA could result in acidification of cytosolic pH. In guard cells, however, metabolic effects of the hormone result in overall alkalinization (Blatt, 2000; Suhita *et al.*, 2004). We measured the effect of ABA on the cytosolic pH of root epidermal cells impaled with pH-sensitive microelectrodes. As indicated in a typical time-course experiment of Fig. 5A, treatment with ABA in the wild type induced, after a lag of about 3min, a small (0.06 pH units) and transient (7min) cytosolic alkalinization followed by a permanent and more pronounced (0.3 pH units) acidification. ABA did not induce cytosolic acidification in either the *wat1-1D* or *112458 pyr/pyl* mutant (Fig. 5A). In the first case, this is because the *wat1-1D* mutant has increased capability for proton extrusion to counteract intracellular acidification (Niñoles *et al.*, 2013) and only the metabolic alkalinization effect of the hormone is observed. The sextuple mutant in PYR/PYL ABA receptors is very insensitive to the hormone, both in guard cells and in other tissues (Gonzalez-Guzmán *et al.*, 2012). This mutant exhibited a cytosolic pH that was more alkaline than that of the wild type in the absence of exogenous ABA, and the addition of the hormone had no effect. The statistical data of the effects of ABA on cytosolic pH of the different genotypes are presented in Fig. 5B.

### ABA hyperpolarizes root epidermal cells by activating K^+^ efflux through the GORK channel

**Fig 6.**
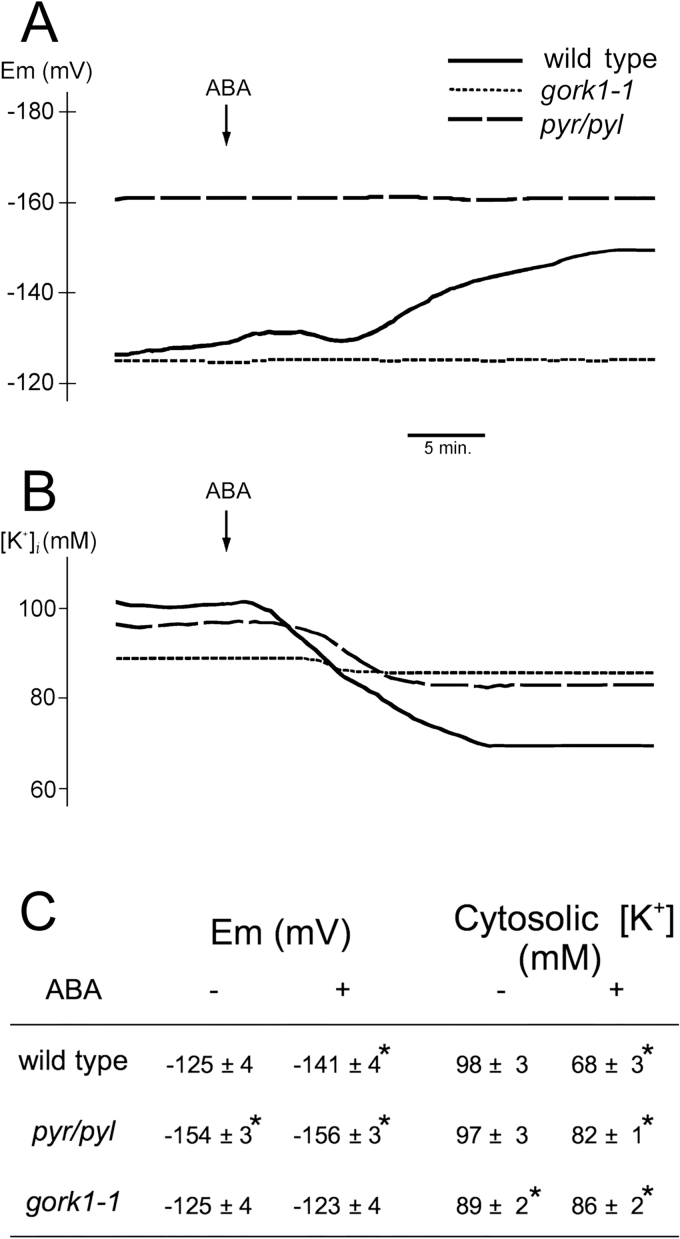
Effect of ABA on membrane electrical potential (Em) and cytosolic [K^+^] in root epidermal cells measured with microelectrodes. (A) Results of a typical experiment for Em determination with the wild type (Col-0 or WS0 gave identical results; solid line), the sextuple knockout in ABA receptors mutant *112458 pyr/pyl* (*pyr/pyl*; dashed line), and the *gork1-1* mutant (punctuated). (B) Results of a typical experiment for [K^+^] determination with the same genotypes. (C) Average of seven (Em) or three ([K^+^] experiments±standard error. * indicates significant differences (*P*<0.01 by Student’s *t*-test) compared with the wild type.

Electrical membrane potential was measured simultaneously with cytosolic pH, and we observed that ABA hyperpolarized root epidermal cells despite inhibiting the electrogenic pump (PM H^+^-ATPase), (Fig. 6A, C). This effect was not observed in the *112458 pyr/pyl* mutant insensitive to ABA. In the absence of exogenous ABA, this mutant was hyperpolarized with respect to the wild type, and this can be explained by the higher H^+^-ATPase activity exhibited by the *112458 pyr/pyl* mutant (see above). This hyperpolarization explains the increased sensitivity to inhibition of germination and seedling establishment by the toxic cations norspermidine and hygromycin B in the *112458 pyr/pyl* mutant (Supplementary Fig. S4 at *JXB* online). The uptake, and therefore the toxicity, of these polycations increased when membrane potential (negative inside) increases (Bissoli *et al.*, 2012). Therefore, results in germinating seeds were in agreement with determinations made in roots.

Membrane electrical potential is determined not only by electrogenic pumps but also by diffusion of ions through the membrane, especially K^+^, Na^+^, and Cl^–^, as predicted by the Goldman equation (Nobel, 1991). A plausible hypothesis for the hyperpolarization induced by ABA, despite inhibiting the H^+^ pump, is that the hormone activates a K^+^ efflux channel and that the K^+^ diffusion potential was greater than the membrane potential before addition of ABA. As indicated in Fig. 6A and C, ABA did not hyperpolarize root epidermal cells in the *gork1-1* mutant defective in the major K^+^ efflux channel (Ache *et al.*, 2000; Hosy *et al.*, 2003). The cytosolic K^+^ concentration (measured by microelectrodes) decreased by 30% after ABA treatment in wild-type *Arabidopsis* but by less than 5% in the *gork1-1* mutant and by only 15% in the *112458 pyr/pyl* mutant (Fig. 6B and C). A final confirmation of K^+^ efflux through the GORK channel was that the decrease in cytosolic K^+^ induced by ABA correlated with an increase in external K^+^ detected with extracellular microelectrodes. This K^+^ efflux was greatly reduced in the *gork1-1* and *112458 pyr/pyl* mutants (Supplementary Fig. S5 at *JXB* online).

Therefore, our data suggested that ABA activates GORK and that the efflux of K^+^ causes hyperpolarization. The internal and external K^+^ concentrations were 98 and 0.1mM, respectively, and therefore the K^+^ diffusion potential was about –180 mV, much higher than the –125 mV measured in the absence of ABA. We investigated whether ABA inhibited K^+^ uptake and whether it caused K^+^ depletion of roots. As indicated in Supplementary Fig. S6 at *JXB* online, ABA inhibited Rb^+^ uptake (as a tracer for K^+^ uptake; Bissoli *et al.*, 2012) by only 20% and there was no significant depletion of total root K^+^ in our time of observation (up to 30min, see legend of Supplementary Fig. S7 at *JXB* online). The observed 30% decrease of cytosolic K^+^ of root epidermal cells (Fig. 6B, C) probably does not translate into significant total K^+^ depletion because of the small (<10%) contribution of the cytosolic compartment compared with the vacuole.

### ABA activates Cl^–^ efflux from root epidermal cells by activating the SLAH3 channel

In stomata guard cells, ABA induces depolarization by both inhibiting the H^+^-ATPase (Merlot *et al.*, 2007) and activating the SLAC1 and SLAH3 anion channels (Vahisalu *et al.* 2008; Geiger *et al.* 2009, 2011). SLAH3 is an SLAC1 homologue that is highly expressed in roots (Zheng *et al.*, 2014), and we have tested the effect of ABA on the roots of the *slah3-1* mutant (Gutermuth *et al.*, 2013). This mutant was hyperpolarized compared with the wild type (–148 versus –125 mV) and ABA still increased the potential by about –15 mV as it did in the wild type (Supplementary Fig. S7). This suggested that the presence of SLAH3 decreased the potential in the wild type and that this anion channel had no effect on the hyperpolarization induced by ABA, which as described above was due to activation of GORK.

**Fig. 7.**
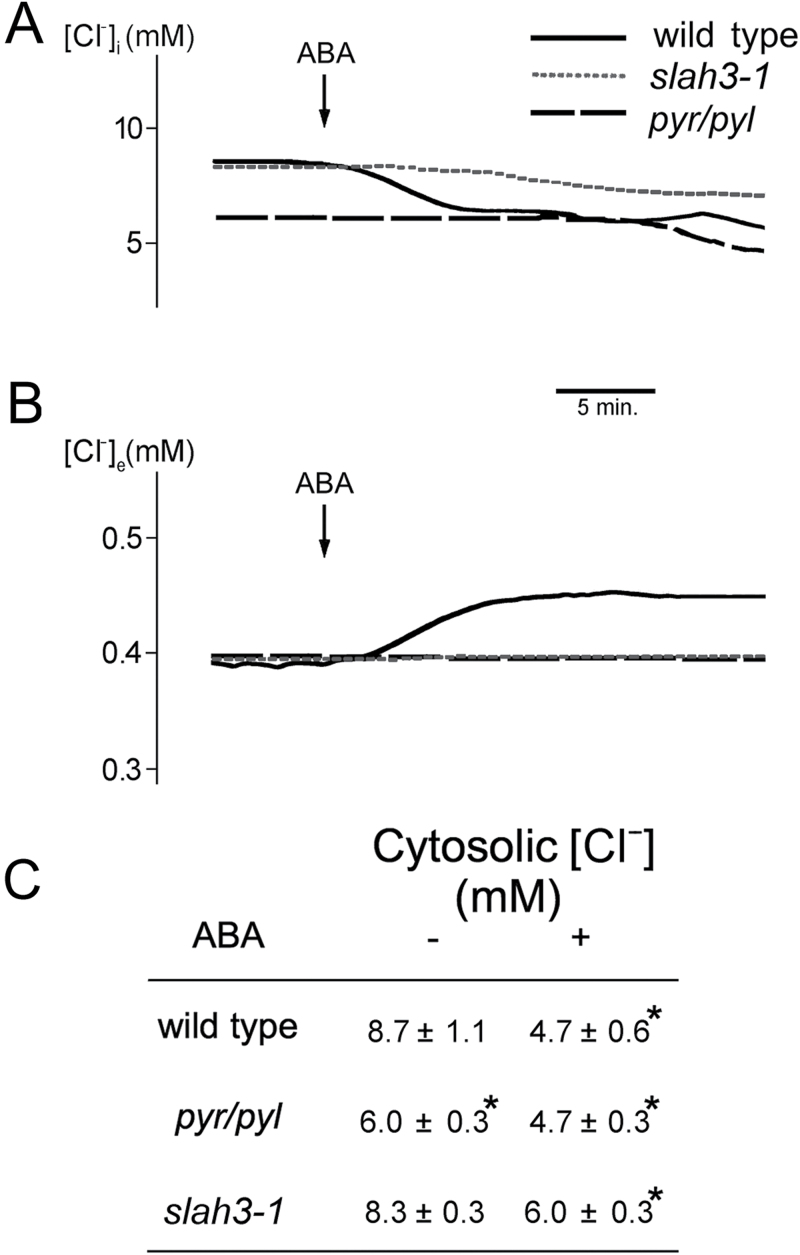
Effect of ABA on chloride concentrations in root epidermal cells measured with microelectrodes. (A) Cytosolic chloride ([Cl^–^]_i_) results of a typical experiment with the wild type (Col-0; continuous line), the sextuple knock-out in ABA receptors *112458 pyr/pyl* (*pyr/pyl*; long dashes), and the *slah3-1* mutant (short dashes). (B) External chloride ([Cl^–^]_e_), Results of a typical experiment with the three genotypes as in (A). (C) Average of three experiments±standard error. * indicates significant differences (*P*<0.01 by Student’s *t*-test) compared with the wild type.

**Fig. 8.**
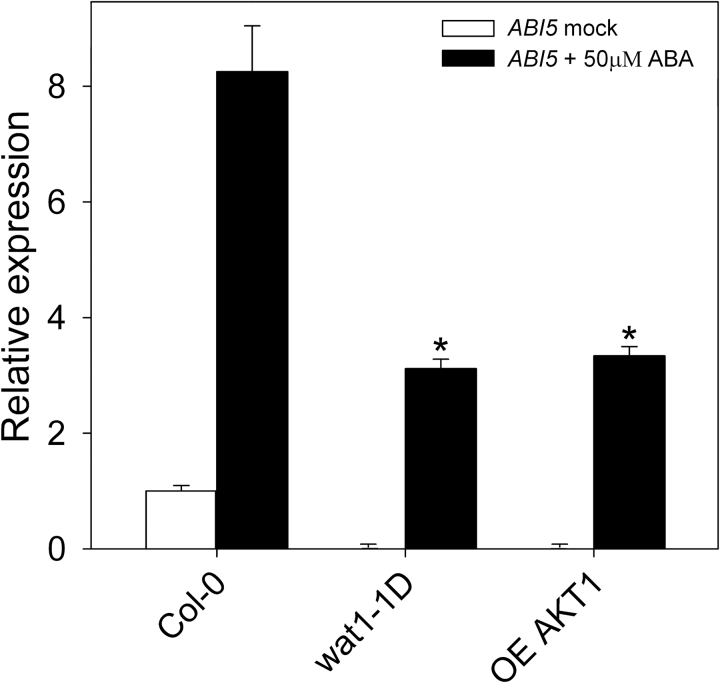
Quantitative real-time PCR analysis of the induction of ABI5 by ABA in germinating seeds of the wild type (Col-0) and the mutants *wat1-1D* and OE *AKT1.* Seeds were placed in MS plates, stratified for 5 d at 4 °C, and germinated for 32h (16h light/8h dark/8h light) at 23 °C. The seeds were then incubated for 30min in liquid MS medium without (white bars) and with (black bars) 50 µM ABA. After RNA extraction and first-strand DNA synthesis with reverse transcriptase, the level of ABI5 cDNA relative to the reference gene *PP2AA3* was determined by real-time PCR as described in Materials and methods. Values are relative to the basal level of the wild type, taken as 1, and are the average of three determinations with error bars corresponding to standard errors. * indicates a significant difference compared with the wild type (*P*<0.01 by Student’s *t*-test).

In order to investigate the effect of ABA on anion transport in root epidermal cells, we measured cytosolic (Fig. 7A, C) and extracellular (Fig. 7B) chloride with Cl^–^-sensitive microelectrodes in different genotypes. In wild-type *Arabidopsis*, ABA induced a fast decrease of cytosolic Cl^–^ during the first 5min followed by a slow decrease that lasted for 30min (Fig. 7A). The first phase correlated with an increase in external Cl^–^, but during the second slow phase there was no increase in external Cl^–^ (Fig. 8B), suggesting vacuolar compartmentation of the anion. The first phase of Cl^–^ efflux was not observed in either the *112458 pyr/pyl* or *slah3-1* mutant, although they exhibited the second slow phase (Fig. 7A, B). Therefore most of the efflux of Cl^–^ induced by ABA was mediated by the PYR/PYL receptors by activating the SLAH3 channel, with the probable vacuolar compartmentation of the slow phase being independent of both systems. In the wild type, the total decrease of cytosolic Cl^–^ induced by ABA after 30min was about 50%, while in *112458 pyr/pyl* and *slah3-1* it was only about 25% (Fig. 7C).

### Transcriptional response to ABA in mutants with improved pH homeostasis

Once we had characterized the effects of ABA on ion homeostasis in roots, we investigated whether the improved pH homeostasis of the *wat1-1D* and OE *AKT1* mutants affected the transcriptional responses to ABA. In germinating seeds, where ABA induces expression of and activates transcription factor ABI5 (Lopez-Molina *et al.*, 2002; Piskurewicz *et al.*, 2008), this induction was greatly reduced (almost 3-fold) in these mutants, and the basal expression level of *ABI5* was also much lower than in the wild type (Fig. 8). On the other hand, in plantlets of 14 d, the induction by ABA of a typical induced gene such as *COR78/RD29A* in the above mutants was not different from the wild type (Supplementary Fig. S8 at *JXB* online). This is in agreement with the observation that in adult plants the inhibition of growth by ABA (Supplementary Fig. S1) and by weak organic acids (Niñoles *et al.*, 2013) is hardly affected by the *wat1-1D* mutation.

## Discussion

In the present work, we have presented evidence for a non-transcriptional mechanism of inhibition of germination and early seedling growth by ABA based on inhibition of PM H^+^-ATPase and a decrease in cytosolic pH, K^+^, and Cl^–^ as determined in *Arabidopsis* roots. The extrapolation from roots to germinating seeds was imposed by technical reasons and its validity is supported by several lines of evidence: (i) mutants with improved pH and K^+^ homeostasis as determined in roots, such as *wat1-1D* and OE *AKT1*, avoid intracellular acidification induced by weak organic acids (such as acetic acid) in root epidermal cells, and their germination and early seedling growth is less inhibited by these acids (Niñoles *et al.*, 2013); (ii) ABA induced cytosolic acidification in root epidermal cells of wild-type *Arabidopsis* but not in the *wat1-1D* mutant (Fig. 6), and OE *AKT1* and *wat1-1D* mutants were tolerant to ABA at the germination and early seedling growth stage (Fig. 1); and (iii) the hyperpolarization of the *pyr/pyl* ABA-insensitive mutant determined with microelectrodes in epidermal root cells (Fig. 7) was also observed indirectly in germinating seeds by the increased sensitivity to toxic cations (Supplementary Fig. S5).

**Fig. 9.**
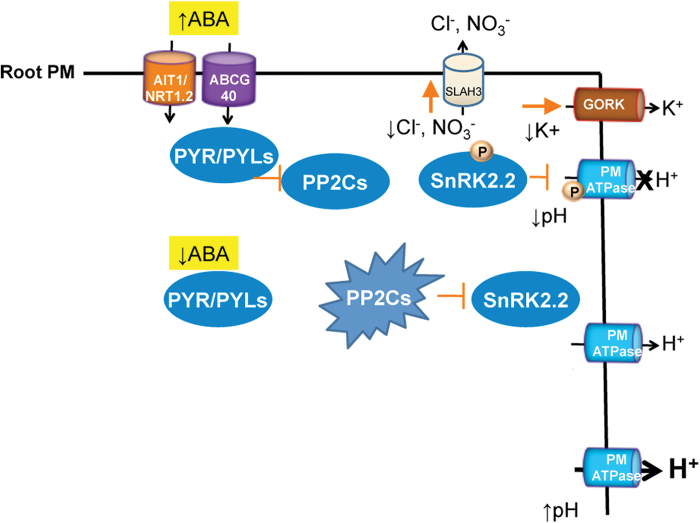
A working model depicting the intracellular acidification induced by ABA in epidermal root cells of *Arabidopsis* and the role of PYR/PYL ABA receptors in pH, K^+^, and Cl^–^ homeostasis. See text for explanation. (This figure is available in colour at *JXB* online.)

A working model for the effects of ABA on *Arabidopsis* roots, and by extrapolation in germinating seeds, is depicted in Fig. 9. The increase of ABA levels leads to cellular uptake by either the ABCG40 (Kang *et al.*, 2010) or AIT1/NRT1.2 (Kanno *et al.*, 2012) systems. This results in inhibition of clade A PP2Cs via PYR/PYL ABA receptors. As a result, SnRK2.2 (and SnRK2.3) is activated and phosphorylates the C-terminal domain of AHA2 H^+^-ATPase at an as yet unidentified residue(s). This results in inhibition of the enzyme and leads to cytosolic acidification, which causes growth inhibition. We have calculated that the decrease in proton motive force by ABA is less than 10 mV (total values about 180 mV, less than 6 % change) and therefore this thermodynamic factor is probably less important for growth inhibition than the kinetic effects of the cytosolic acidification of 0.4 pH units (a 2.5-fold increase in H^+^ concentration; Fig. 5). ABA also promotes K^+^ efflux via the GORK channel and Cl^–^ efflux via SLAH3. Nitrate efflux probably also occurs via SLAH3 (Roelfsema *et al.*, 2012) and this may have nutritional consequences. The efflux of K^+^, Cl^–^, and other anions will result in loss of turgor, and this may also contribute to growth inhibition. At basal ABA levels, clade A PP2Cs are active and inhibit SnRK2.2 (and SnRK2.3) activity, which prevents inhibitory phosphorylation of the C-terminal domain of AHA2 H^+^-ATPase. In the *112458 pyr/pyl* mutant, PP2Cs are hyperactive at endogenous ABA levels and promote enhanced H^+^-ATPase activity compared with wild type, which results in cytosolic alkalinization and hyperpolarization. Therefore, PYR/PYLs regulate pH homeostasis both under non-stress and stress conditions. The altered pH and electric potential found in roots of the *112458 pyr/pyl* mutant might explain the root growth impairment observed previously in strongly ABA-insensitive mutants (Gonzalez-Guzmán *et al.*, 2012).

This ion homeostasis mechanism of ABA action in germinating seeds may be complementary to the transcriptional mechanism based on induction and activation of transcription factor ABI5, which in turn induces expression of genes encoding inhibitors of the cell cycle and metabolism as discussed in the Introduction. In fact, we made the interesting observation that induction by ABA of ABI5 was greatly reduced in germinating seeds of the *wat1-1D* and OE *AKT1* mutants (Fig. 8). Therefore, the cytosolic acidification induced by ABA seems important to obtain the full transcriptional response to the hormone at this stage of growth. As ABI5 is important for the inhibition of growth by ABA in germinating seeds, a transcriptional mechanism modulated by cytosolic acidification is probably operative. This would act in parallel to direct growth inhibitory effects of decreased PM H^+^-ATPase, cytosolic pH, K^+^ and anions. These results support the role of cytosolic pH as signal and messenger in plant cells (Felle, 2001). Although pH affects most proteins, only some may act as pH sensors by crucial conformational changes such as the yeast Hal3-Ppz1 protein phosphatase (Yenush *et al.*, 2005).

The mutants with improved pH homeostasis utilized in the present work exhibited similar inhibition of growth by ABA in adult plants as the wild type (Supplementary Fig. S1). Also, transcriptional responses to ABA were normal in adult roots (Supplementary Fig. S8). As the changes in pH, K^+^, and Cl^–^ homeostasis induced by ABA were measured in mature roots, we have to postulate that seed germination and early seedling growth are particularly sensitive to decreases in cytosolic pH, K^+^, and anions. As discussed above, a plausible mechanism to explain this singularity is the pH-dependent induction by ABA of ABI5 in germinating seeds. The existence of a pH-dependent mechanism operating upstream of the ABI5 transcriptional response is currently under investigation.

The inhibition of root PM H^+^-ATPase by ABA identified in the present work requires the known ABA receptors and downstream protein kinases and phosphatases, but the detailed mechanism is unknown. At variance with regulation of the enzyme by ABA in guard cells (Yin *et al.*, 2013) and hypocotyls (Hayashi *et al.*, 2014), phosphorylation of the known activating site (Trh947) of AHA2 H^+^-ATPase was not affected by ABA in our root experimental system (Supplementary Fig. S2). We could demonstrate *in vitro* specific phosphorylation of the C-terminal domain of AHA2 H^+^-ATPase by SnRK2.2 but not by SnRK2.6/OST1, the kinase specific for guard cells (Fig. 4B). We could not identify the phosphorylation site by either phosphoproteomics (Navajas *et al.* 2011) or mutagenesis of several candidate residues such as Ser899, Ser904, Ser931, and Thr924 (Joshi-Saha *et al.*, 2011; Rudashevskaya *et al.*, 2012). There are two examples of inhibitory sites in AHA2 H^+^-ATPase: Ser899 is an inhibitory site upon phosphorylation triggered by flagellin (Nühse *et al.*, 2007) and by a RALF peptide hormone (Haruta *et al.*, 2014), and Ser931 is an inhibitory site upon phosphorylation by calcium-activated heteromeric protein kinase PKS5/CIPK11-SCaBP1/CBL2 triggered at high external pH (Fuglsang *et al.*, 2007). Accordingly, a plausible mechanism for the effect of ABA on root PM H^+^-ATPase is that the enzyme is phosphorylated by SnRK2.2 (and probably SnRK2.3) at a novel inhibitory site within the C-terminal domain. There are six more putative phosphorylation sites in the C-terminal domain of H^+^-ATPase that have not yet been mutagenized (Fuglsang *et al.*, 2007).

The effects of ABA in *Arabidopsis* roots described in the present work resemble the situation in guard cells (Kim *et al.*, 2010; Hedrich, 2012) but there are important differences. In both systems, there is inhibition of H^+^-ATPase and activation of GORK and SLAH3, but the major differences are in the mechanism of activation of the H^+^-ATPase (as described above) and in the direction of changes in cytosolic pH and membrane potential. In guard cells, ABA induces cytosolic alkalinization, instead of the acidification observed in roots, and this alkalinization is an important signal for stomata closure (Blatt, 2000; Suhita *et al.*, 2004). This occurs because in guard cells ABA activates the gluconeogenic conversion of malate into starch, a metabolic reaction that involves decarboxylation (R-COO^–^+H^+^→R-H+CO_2_) and therefore alkalinization (Davies, 1986). This also serves to reduce intracellular osmotic concentrations together with the efflux of K^+^ and anions (MacRobbie, 1998). Apparently, this metabolic alkalinization is less important in roots, although it can be observed as a small transient pH increase upon ABA addition, followed by permanent acidification (Fig. 6A). Concerning membrane potential, ABA depolarizes in guard cells but hyperpolarizes in root epidermal cells (Fig. 7). Although in both cases the inhibition of the H^+^-ATPase favours depolarization, this is reinforced by anion efflux in guard cells, while in roots it is counteracted by K^+^ efflux through the GORK channel. Apparently, in *Arabidopsis* root epidermal cells, anion channels are less important than in guard cells, while GORK activity is higher.

## Supplementary data

Supplementary data is available at *JXB* online.

Supplementary Fig. S1. Plantlet growth is slightly less sensitive to ABA in the *wat1-1D* mutant than in wild type (Col-0).

Supplementary Fig.S2. ABA decreases neither the level of plasma membrane H^+^-ATPase nor the phosphorylation of Thr947.

Supplementary Fig. S3. The C-terminal domain of AHA2 with the double mutation Thr924Ala Ser931Ala is phosphorylated by SnRK2.2 as well as wild type.

Supplementary Fig. S4. Mutants with reduced sensitivity to ABA are hypersensitive to toxic cations.

Supplementary Fig. S5. Direct measurement with external microelectrodes of ABA-induced K^+^ efflux from root epidermal cells of *Arabidopsis* wild type (Col 0), *112458 pyr/pyl* mutant (sextuple), and *gork1-1* mutant.

Supplementary Fig. S6. Inhibition by ABA of rubidium uptake, as a tracer of K^+^ transport, in *Arabidopsis* (Col-0) roots.

Supplementary Fig. S7. Effect of ABA on the membrane potential of root epidermal cells from the wild type (Col 0) and *slah3-1* mutant (slah).

Supplementary Fig. S8. Induction of COR78/RD29A by ABA in roots is the same in wild type (Col-0) and *wat1-1D* and OE *AKT1* mutants.

Supplementary Table S1. Primers utilized in the present work.

Supplementary Data

## Abbreviations:

ABA: abscisic acid
Arabidopsis: *Arabidopsis thaliana*
PP2C: protein phosphatase type 2C
PM: plasma membrane
PYR/PYL/RCAR: PYRABACTIN RESISTANCE1 /PYR1-LIKE/ REGULATORY COMPONENTS OF ABA RECEPTORS.
