# Supplementary material for: A mechanism of growth inhibition by abscisic acid in germinating seeds of Arabidopsis thaliana based on inhibition of plasma membrane H+-ATPase and decreased cytosolic pH, K+, and anions
Source: J Exp Bot. 2014 Nov 4;66(3):813–25. doi: 10.1093/jxb/eru442 (PMC4321545; doi:10.1093/jxb/eru442)
Supplement: Supplementary Data [file supp_66_3_813__index.html]

A mechanism of growth inhibition by abscisic acid in germinating seeds of Arabidopsis thaliana based on inhibition of plasma membrane H+-ATPase and decreased cytosolic pH, K+, and anions — A mechanism of growth inhibition by abscisic acid in germinating seeds of Arabidopsis thaliana based on inhibition of plasma membrane H+-ATPase and decreased cytosolic pH, K+, and anions — Supplementary Data 

# A mechanism of growth inhibition by abscisic acid in germinating seeds of *Arabidopsis thaliana* based on inhibition of plasma membrane H+-ATPase and decreased cytosolic pH, K+, and anions

## Supplementary Data

Data files

**Files in this Data Supplement:**

- Supplementary Data - Supplementary Data
