# Supplementary material for: A mechanism of growth inhibition by abscisic acid in germinating seeds of Arabidopsis thaliana based on inhibition of plasma membrane H+-ATPase and decreased cytosolic pH, K+, and anions
Source: J Exp Bot. 2014 Nov 4;66(3):813–25. doi: 10.1093/jxb/eru442 (PMC4321545; doi:10.1093/jxb/eru442)
Supplement: Supplementary Data [file supp_eru442_jexbot135244_file001.pdf]

SUPPLEMENTARY MATERIAL FOR ONLINE-ONLY PUBLICATION:

A mechanism of growth inhibition by abscisic acid in germinating seeds of *Arabidopsis thaliana* based on inhibition of plasma membrane H^+-ATPase and decreased cytosolic pH, K^+ and anions in roots

M. Dolores Planes[1,*], Regina Niñoles[1,*], Lourdes Rubio[2,*], Gaetano Bissoli[1,*], Eduardo Bueso[1], María J. García-Sánchez[2], Santiago Alejandro[1], Miguel Gonzalez-Guzmán[1], Rainer Hedrich[3], Pedro L. Rodriguez[1] José A. Fernández[2] and Ramón Serrano[1,$]

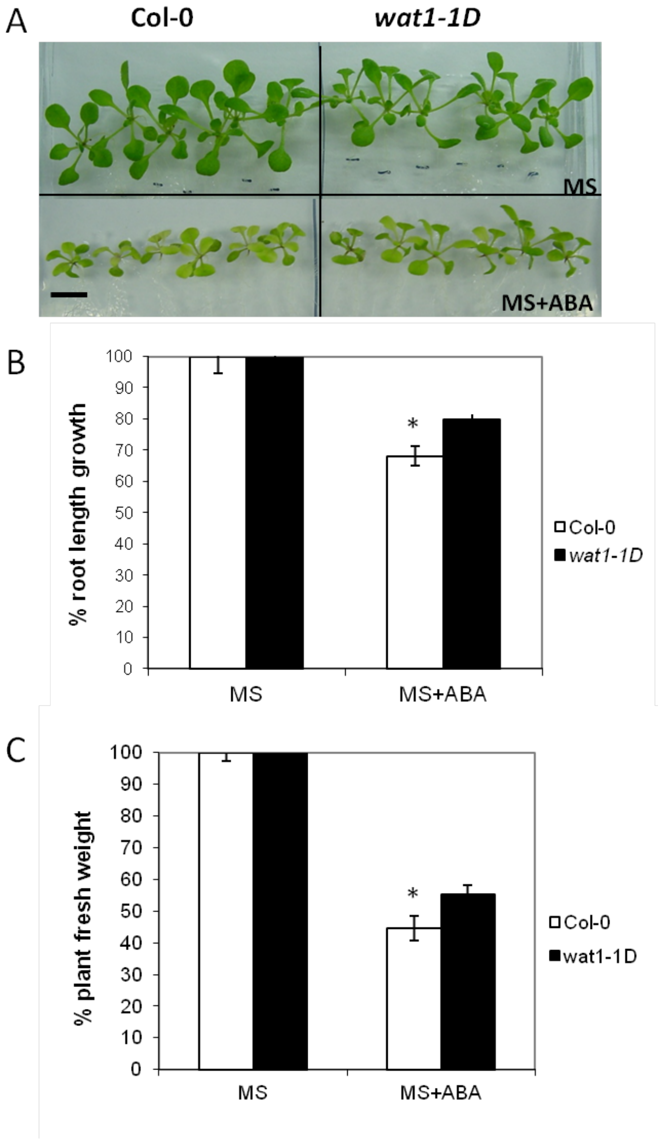

Fig. S1. Plantlet growth is slightly less sensitive to ABA in the *wat1-1D* mutant than in wild type (Col-0). Plantlets were germinated and grown in vertical plates for 7 days and then transferred to fresh plates without and with 10 µM ABA as indicated. A: visual result of a typical experiment after 8 days of growth. Scale bar corresponds to 1 cm. B: increase in root length during 3 days, relative values. Absolute values in the absence of ABA were 2.7 ± 0.1 (Col-0) and 2.2 ± 0.2 (*wat1-1D*) cm. C: plant fresh weight after 8 days, relative values. Absolute values in the absence of ABA were 30.1 ± 0.6 (Col-0) and 26.0 ± 0.6 (*wat1-1D*) mg per plant. * indicates significant (P < 0.05) differences between Col-0 and *wat1-1D* by Student´s test.

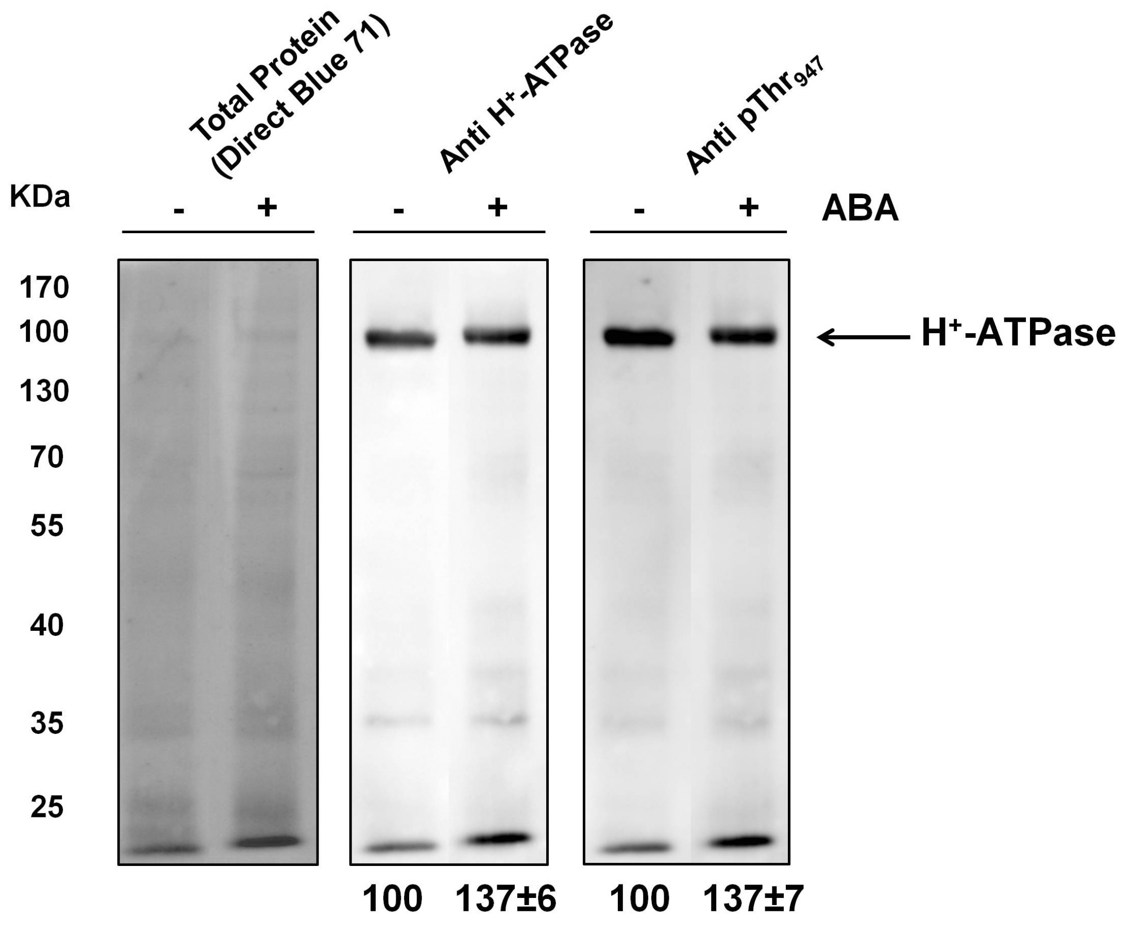

Fig. S2. ABA decreases neither the level of plasma membrane H^+-ATPase nor the phosphorylation of Thr947. Results of a typical experiment are shown. The blot was first decorated with the antibody against the phosphorylated peptide, stripped by incubation 1 h in 0.2 M glycine-HCl pH 2.7, decorated with antibody against the H^+-ATPase, and finally stained for total protein with Direct Blue 71. Numbers below indicate the quantification of the H^+-ATPase band by ImageJ in 4-5 experiments as average of relative values (+ABA/-ABA in %) with the standard error.

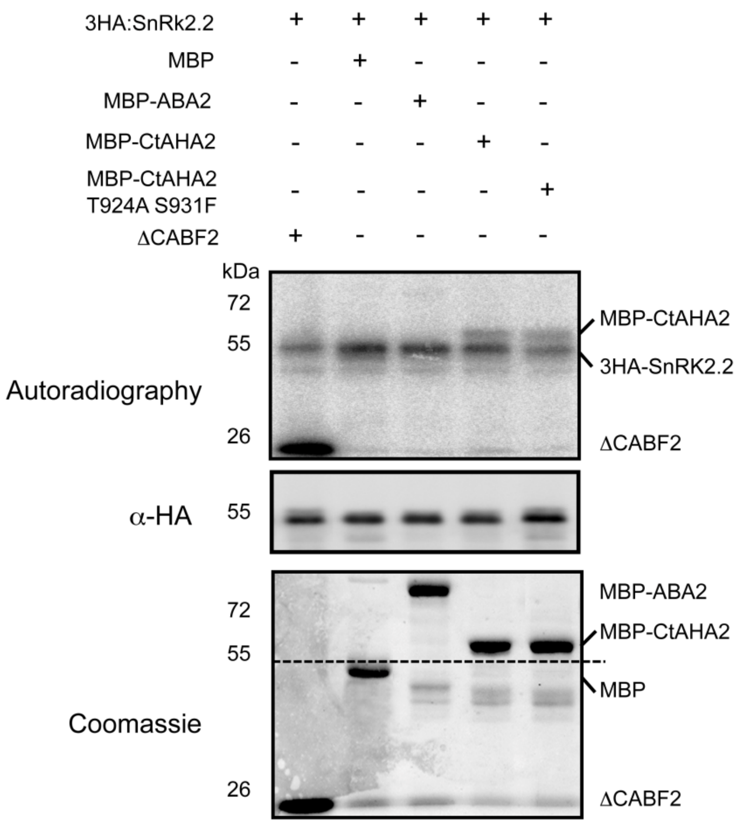

Fig. S3. The C-terminal domain of AHA2 with the double mutation Thr924Ala Ser931Ala is phosphorylated by SnRK2.2 as well as wild type. The first three lanes from the left indicate that 3HA-SnRK2.2 phosphorylates itself and its known substrate ΔCABF2 (without carboxy terminal and tagged with 6xHis at the N-terminus; Dupeaux et al., 2011) but not MBP (maltose binding protein) or MBP-ABA2. The last 2 lanes indicate that HA-SnRK2.2 phosphorylates itself and, to a similar extent, the wild type C-terminus of AHA2 H^+-ATPase and a mutated version containing a double T924A S931A mutation. The dashed line in the Coomassie staining panel indicates the migration of the immunoprecipitated 3HA-SnRK2.2, which was estimated by overlapping with the anti-HA immunoblot.

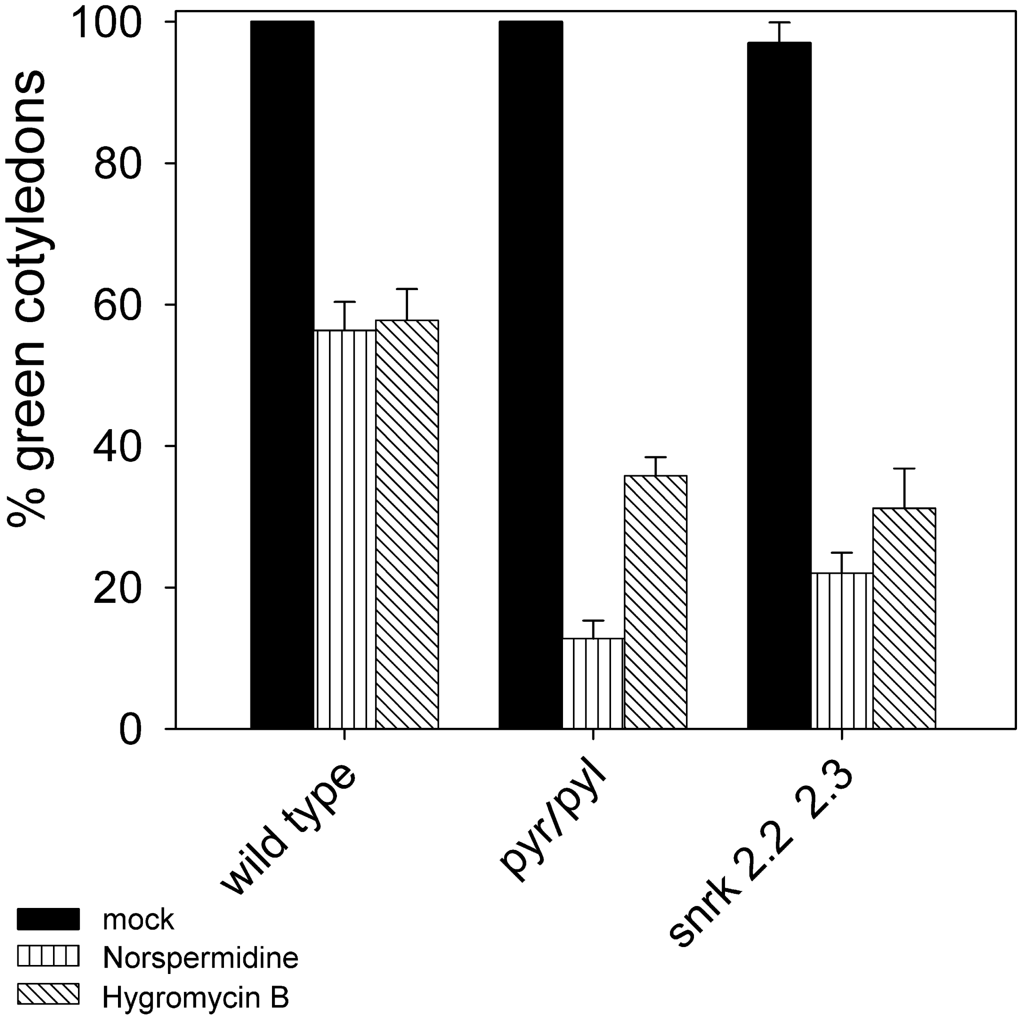

Fig. S4. Mutants with reduced sensitivity to ABA are hypersensitive to toxic cations. The sextuple ABA receptor mutant (*pyr/pyl*) and the double protein kinase mutant (*snrk2.2 snrk2.3*) are more inhibited by than wild type (Col-0) by the toxic cations norsperimidine and hygromycin B during germination and seedling establishment (appearance of green cotyledons 7 days after sowing). Results are the average of three determinations and error bars correspond to standard errors. Differences with wild type in the presence of toxic cations are statistically significant with $p < 0.01$ by Student test.

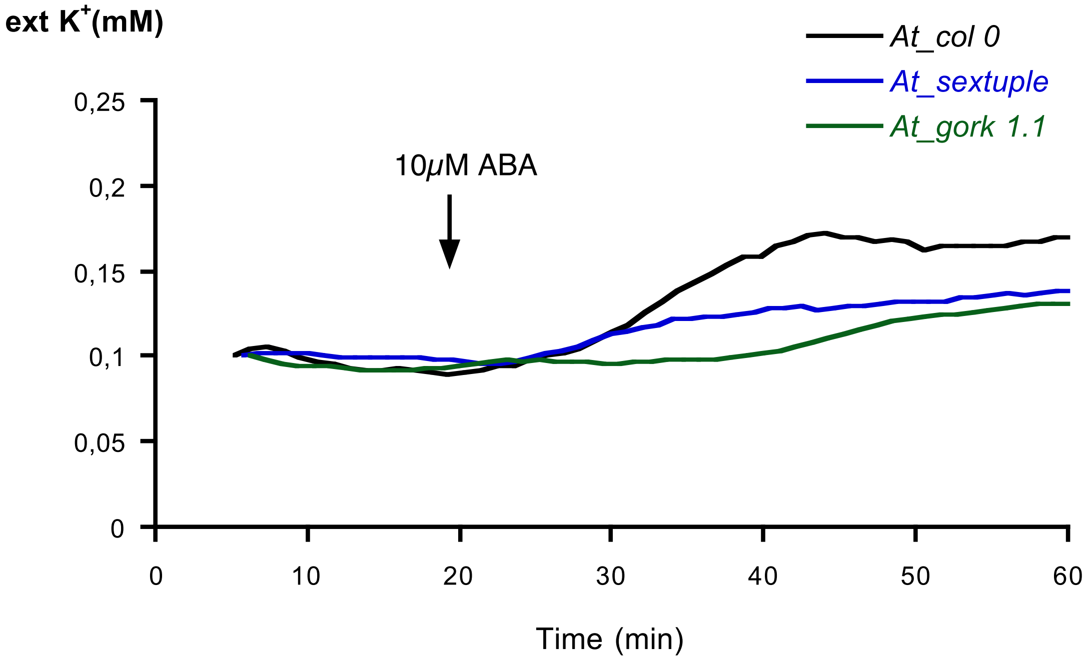

Fig. S5. Direct measurement with external microelectrodes of ABA-induced K^+ efflux from root epidermal cells of Arabidopsis wild type (Col 0), *112458 pyr/pyl* mutant (sextuple) and *gork1-1* mutant. The experiment was repeated three times with similar results.

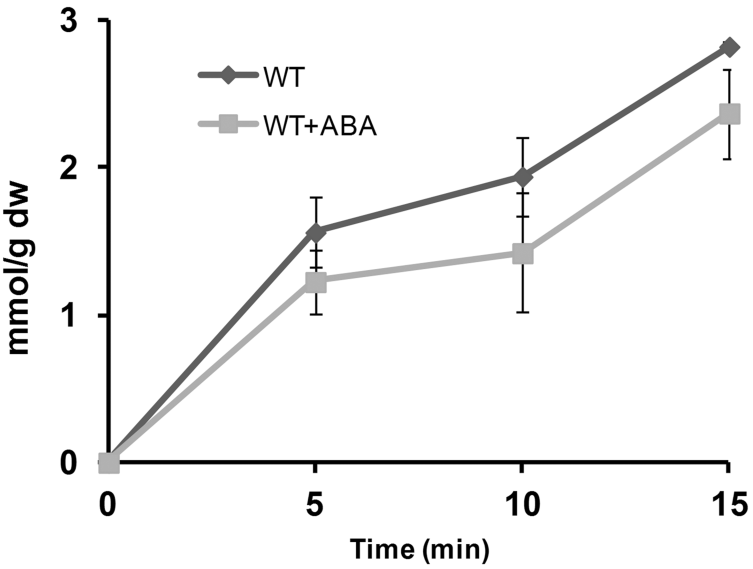

Fig. S6. Inhibition of rubidium uptake, as tracer of K^+ transport, in Arabidopsis (Col-0) roots. The method was as described by Alejandro et al. (2007). Basically, seedlings were germinated and grown in potassium-free liquid medium for 15 days, and then incubated in the same medium containing 2 mM RbCl. Samples were taken at the indicated times, washed, roots were separated, extracted with nitric acid, centrifuged, and the Rb^+ and K^+ content in the supernatant was determined by atomic absorption spectrophotometry. The total K^+ content of roots did not change during the time course of the experiment and up to 30 min and it was 620 ± 12 mmol/g dw of roots (dw, dry weight).

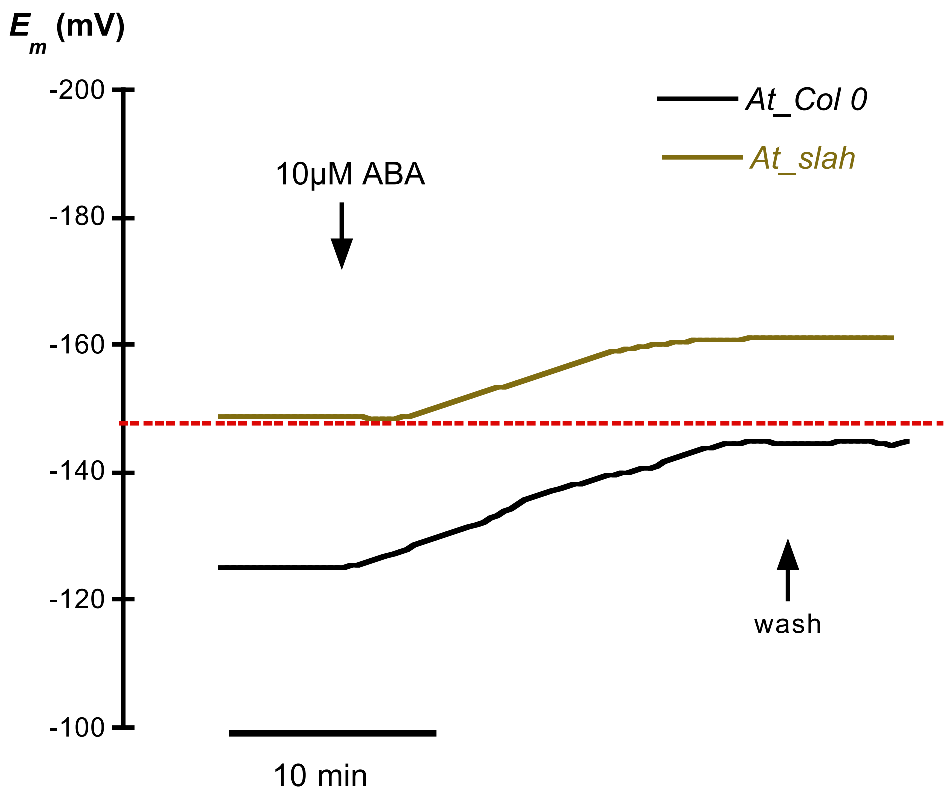

Fig. S7. Effect of ABA on the membrane potential of root epidermal cells from the wild type (Col 0) and *slah3-1* mutant (slah). The experiment was repeated three times with similar results.

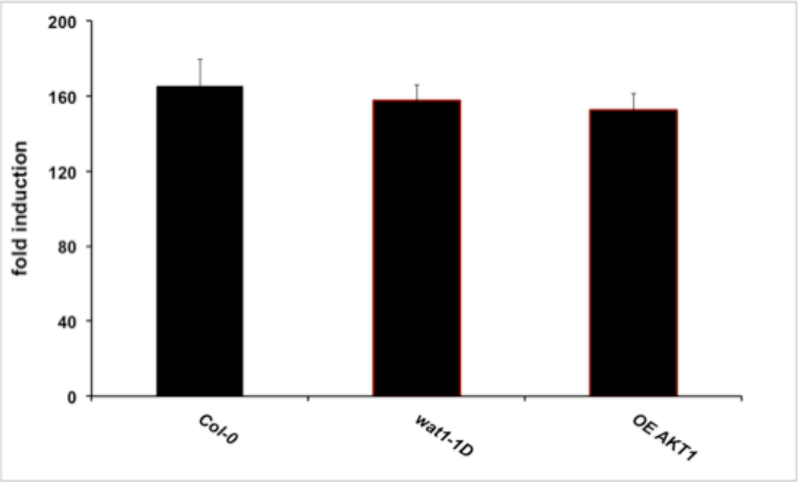

Fig. S8. Induction of COR78/RD29A by ABA in roots is the same in wild type (Col-0) and *wat1-1D* and OE*AKT1* mutants. Plants were grown for 2 weeks in vertical plates and then transferred to liquid medium and incubated without or with 50 µM ABA during 90 min. COR78/RD29A mRNA was quantified by reverse transcription and Real Time-PCR as described in methods using primers COR78-F and COR78-R (Supplemental Table 1). Fold induction is the average of three experiments and error bars correspond to the standard error. The reference gene was ACT8.

Table S1. Primers utilized in the present work. Ending F and R indicate forward and reverse primers.

Name	Sequence (5'->3')	Underlined site
AHA2Ct-F	CCGGAATTCCGATACATCTTGAGCGGAAAG	*EcoR* I
AHA2Ct-R	AAACCGAAGCTTCTACACAGTGTAGTGACTGGGAG	*Hind* III
S899P-F	CATCTTCCCTGAGAAAGGGCCCTACAGAGAATTGTCTGAGATCGC	*Bsp*120I
S899P-R	GCGATCTCAGACAATTCTCTGTAGGGCCCTTTCTCAGGGAAGATG	*Bsp*120I
S904L-F	GGAAGTTACAGAGAATTGCTCGAGATCGCT GAGCAAGCTAAG	*Xho* I
S904L-R	CTTAGCTTGCTCAGCGATCTCGAGCAATTCTCTGTAACTTCC	*Xho* I
S931F-F	CACTCAAGGGACATGTGGAATTCGTCGTGAAGCTAAAGGGCTTGG	*EcoR* I
S931F-R	CCAAGCCCTTTAGCTTCACGACGAATTCCACATGTCCCTTGAGTG	*EcoR* I
T924A-F	AGGCTTAGGGAGCTGCACGCTAGCAAGGGACATGTGGAATCAGTC	*Nhe* I
T924A-R	GACTGATTCCACATGTCCCTTGCTAGCGTGCAGCTCCCTAAGCCT	*Nhe* I
F3g50500	ATGGATCCGGCGACTAATTCA	
R3g50500	TTTGTCGACTCAGAGAGCATAAACTATCT	
ABI5-F	TAGTGGATGGTCCAGTGGAGA	
ABI5-R	CCAACTCCGCCAATGCATG	
PP2AA3-F	ACCTGCGGTAATAACTGCATCTA	
PP2AA3-R	CCGAACATCAACATCTGGGTC	
COR78-F	CAGTCCAAAGTTACTGATCCC	
COR78-R	TTCCAGAATCGTTCTTCTGATC	
ACT8-F	CATGGAGAAGATTTGGCATCAC	
ACT8-R	GAGGATAGCATGTGGAAGTGA	
